# Integration of meta-analysis, machine learning and systems biology approach for investigating the transcriptomic response to drought stress in *Populus* species

**DOI:** 10.1038/s41598-023-27746-6

**Published:** 2023-01-16

**Authors:** Ahmad Tahmasebi, Ali Niazi, Sahar Akrami

**Affiliations:** Institute of Biotechnology, Shiraz University, Shiraz, 7144165186 Iran

**Keywords:** Plant molecular biology, Plant stress responses, Plant sciences

## Abstract

In *Populus*, drought is a major problem affecting plant growth and development which can be closely reflected by corresponding transcriptomic changes. Nevertheless, how these changes in *Populus* are not fully understood. Here, we first used meta-analysis and machine learning methods to identify water stress-responsive genes and then performed a systematic approach to discover important gene networks. Our analysis revealed that large transcriptional variations occur during drought stress. These changes were more associated with the response to stress, cellular catabolic process, metabolic pathways, and hormone-related genes. The differential gene coexpression analysis highlighted two acetyltransferase *NATA1*-like and putative cytochrome P450 genes that have a special contribution in response to drought stress. In particular, the findings showed that MYBs and MAPKs have a prominent role in the drought stress response that could be considered to improve the drought tolerance of *Populus*. We also suggest *ARF2*-like and *PYL4*-like genes as potential markers for use in breeding programs. This study provides a better understanding of how *Populus* responses to drought that could be useful for improving tolerance to stress in *Populus*.

## Introduction

Climate change is a worldwide problem that has negative impacts on ecological systems, species distribution and forest growth. High temperatures resulting from climate change enhance the frequency and intensity of drought. Such extreme conditions of drought can lead to decrease forest productivity and increase tree mortality^1–3^. Drought has significant effects on plant growth and development; these effects are mainly due to changes in the normal rates of photosynthesis, osmotic adjustment and oxidative damage^4,5^. Response of plants to drought is a complex mechanism involving morphological, physiological, and metabolic changes^6^. A variety of protective genes, proteins, and pathways are involved in the protective responses against stress conditions^7^.

Poplars (*Populus* sp.) are trees with high ecological and economic values, which are widely distributed around the world and are known as sensitive woody plants to water^8^. *Populus* is serves as a model system for biology research and analysis of genetics in forest trees^9^. Genomic information for the *Populus* genus provides a valuable resource for investigating genome features and the characterization of stress-related genes in woody plants. Drought stress can cause damage to cellular structures and macromolecules, decreasing the photosynthesis rate, the reduction of growth and biomass production, and sometimes even lead to tree death. Studies have shown that at the molecular level, several genes and multiple biological processes are involved in the adaptation of *Populus* to stress. For example, water deficit significantly changes expression profiles of numerous genes encoding transcription factors (TFs), plant phosphatases and hormones^10^. Additionally, several genes such as *ProDH*, *JAZ3* and *RAP2*.*6*, *LEA* and *ABA1* have been induced that play an important role in the response to drought stress in *Populus*^11,12^. Investigating the transcriptome and discovering different drought-tolerant genes are greatly informative for understanding the mechanisms of plant stress tolerance. Different strategies have been employed to determine the molecular basis of *Populus* response to drought stress^4,13–15^.

In recent years, high throughput gene expression technologies provide valuable information about transcriptome for research on genes and molecular mechanisms involved in stress. The detection of differentially expressed genes is one of the strategies for data analysis. A wide variety of methods is greatly performed on the dataset with small sample sizes for the screening of genes. Restricting the analysis to an individual study reduces statistical power which may lead to unreliable results. Meta-analysis is a powerful approach for integrating gene expression datasets and obtaining gene signatures more robust and accurate and through increasing sample size. Several studies have used meta-analysis for the identification of responsive genes environmental stress in plants^16–19^. Recently, machine learning models are known as an attractive strategy to gain new biological insights. Generally, these methods with efficient dimensionality reduction of the data and feature selection methodologies can integrate transcriptome studies and as a result, find the significant features and underlying mechanisms^20^. Different algorithms such as support vector machine (SVM) and principal component analysis (PCA) have been employed in feature selection. These algorithms use various evaluation criteria for classifying data and scoring the input features. In the area of plant stress, the effective use of machine learning and feature selection models for selecting gene features is reported in rice^21^, Arabidopsis^22^, potato^23^, and maize^24^. However, at the transcriptome level, the machine learning algorithms for identifying key signatures related to environmental stress have not been applied in *Populus*.

Despite the importance of differentially expressed genes identification, this strategy mostly focuses on the discovery of gene contents and suffers from exploring relationships among genes. Coexpression network analyses allow us to obtain a system-level view of gene–gene connections. Moreover, the biological role of genes with unknown functions can be predicted using methods based on coexpression^25^. There are different approaches including coexpression and differential coexpression analyses for constructing from gene expression data. The coexpression builds networks of genes based on the similarity between expression patterns of the gene pair across all the samples, where can be used for identifying co-activate and regulatory genes. While differential coexpression analysis discovers genes with altered coexpression partners under different states which can lead to defining gene groups affected by the change of state^26^. These analyses have been widely applied to better understand the molecular mechanisms in plant species^16,27^.

In this study, we employed large transcriptome data to gain a comprehensive view of drought stress response in *Populus*. We performed a meta-analysis in combination with machine learning techniques to identify drought-responsive genes. Additionally, through coexpression network analyses, we detected functional sets of genes associated with drought stress. Our findings can provide valuable information about the underlying mechanisms related to a drought stress response which can be used for future genetic improvement and breeding programs in tree species.

## Results

**Figure 1:**
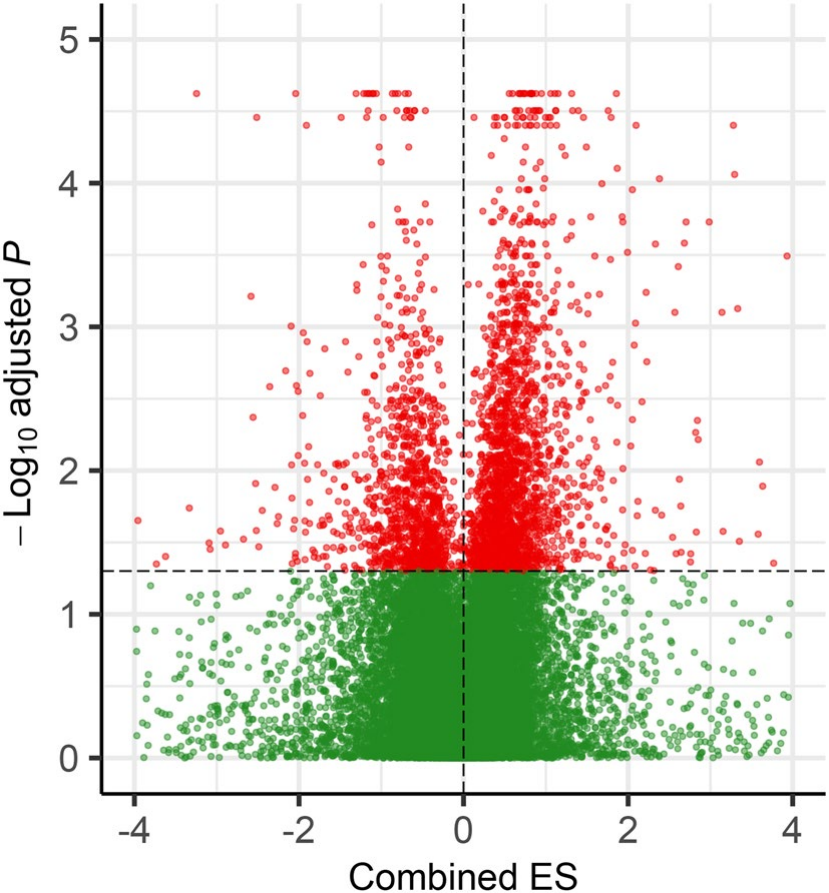
Gene expression comparison between normal and stress conditions. A volcano plot showing combined effect size (x-axis) and significance level (− log_10_-adjusted *p*-value; y-axis) for genes differentially expressed between normal and stress samples. The significant up and down-regulated genes are plotted as red dots.

**Figure 2:**
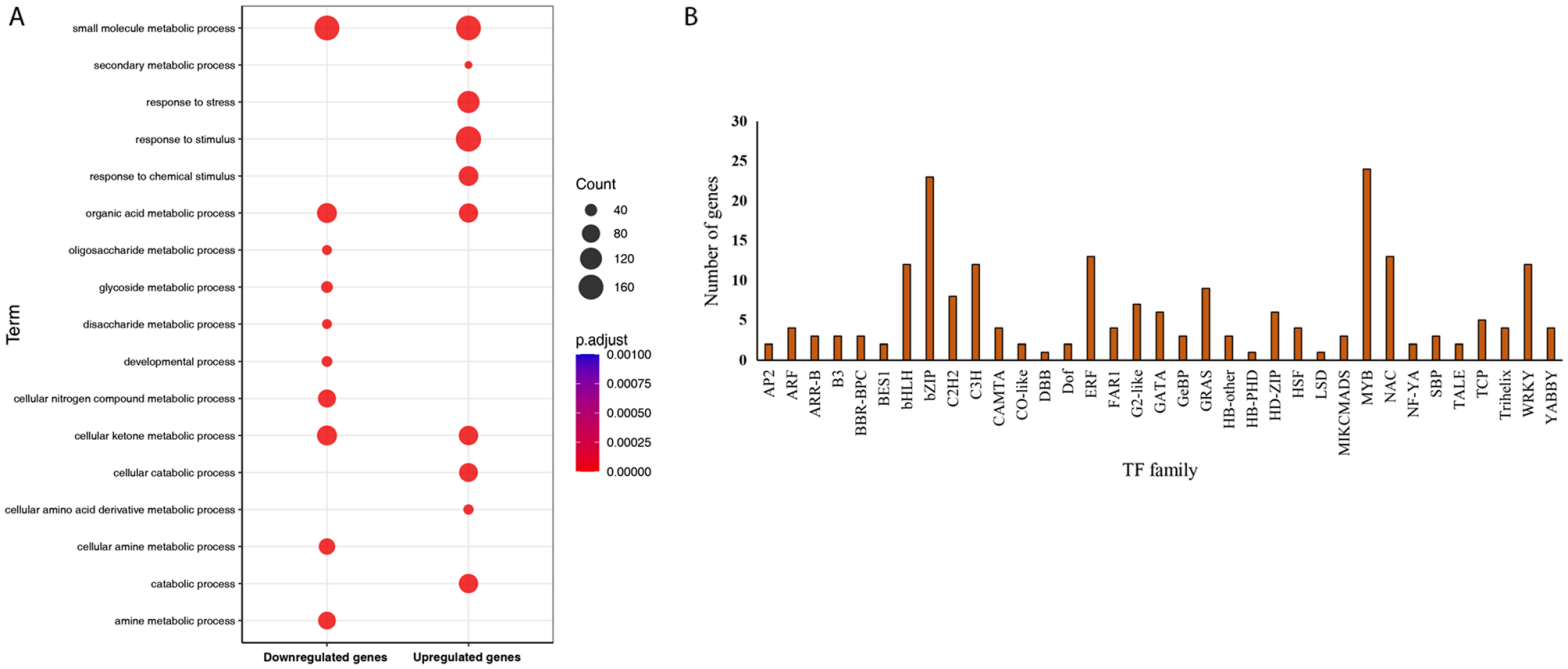
(**A**) Top 10 gene ontology biological processes (GO-BP) terms of the up-regulated and the down-regulated differentially expressed genes (DEGs) (**B**) Distribution of transcription factor (TF) families in the differentially expressed genes (DEGs). The number of genes is shown for each transcription factor family on the y-axis.

To understand the transcriptional responses of *Populus* to drought stress, 13 microarray datasets (Supplementary Table S1) consisting of 324 arrays in total were considered. After pre-processing and removing the batch effect, the normalized datasets were obtained for further downstream analysis. These datasets were divided into control and stress conditions in each study. From meta-analysis, we identified 3178 genes with differences in expression level between stress and control conditions (FDR < 0.05). Among DEGs, 2060 genes were up-regulated and 1118 genes were down-regulated in drought compared to normal conditions (Supplementary Table S2, Fig. 1). The gene ontology (GO) terms showed that upregulated DEGs were most significantly enriched in response to stress, response to stimulus and cellular catabolic process while the downregulated DEGs were related to small molecule metabolic process, cellular ketone metabolic process and organic acid metabolic process (Supplementary Table S3, Fig. 2A). The most important molecular function terms associated with up-regulated DEGs included phosphoprotein phosphatase activity and phosphoric ester hydrolase activity. Moreover, results of KEGG pathway analysis revealed that the up-regulated DEGs were mostly enriched in metabolic pathways, spliceosome and plant hormone signal transduction, while the down-regulated DEGs were significantly associated with phagosome and amino sugar and nucleotide sugar metabolism (Supplementary Table S3).

Due to the high dimensionality of microarray gene expression data, feature selection techniques as a branch of machine learning can be of great help in distinguishing genes with key biological functions. In this study, to discover the transcriptomic signature of response to drought stress in poplar species, eight feature selection models were implemented on the gene expression dataset in stress and normal groups. In total, 648 genes were identified as the most important features by at least one of the models (Supplementary Table S4). Functional annotation showed that the feature genes enriched in response to abiotic stimulus. In addition, a lot of genes were related to secondary metabolic process. Interestingly, the seven methods selected *Auxin response factor 2*-like (PtpAffx.211941.1.S1_at) and *PYL4*-like (PtpAffx.31936.1.A1_at) as important features. Moreover, the meta-analysis and feature selection techniques had 232 common genes among which the genes, 6 genes are involved with the MAPK signaling pathway. Thus, our results indicate key role of MAPKs in *Populus* during drought stress. Here, we also identified 210 transcription factors from 35 different gene families responding to drought stress. Among them, the MYB, bZIP, NAC, ERF, and bHLH families comprise a high proportion of drought-responsive members (Fig. 2B).

### *Cis*-regulatory element analysis

To discover the conserved motifs and consensus *cis*-regulatory elements (CREs) in the promoters of DEGs, we applied the MEME tool and identified 14 motifs with lengths ranging from 15 to 50aa (Table 1). We also compared the identified motifs with known motifs in the CIS-BP database. We found that eight of the motifs were matched to the known motifs related to various TFs, including ARID, C2H2, AHL, AP2/B3-like, BBR/BPC, MYB, and Sox (Supplementary Table S5). GO term analysis for motifs revealed that motifs are involved in the regulation of transcription, transmembrane receptor protein tyrosine kinase signaling pathway and development (Table 1, Supplementary Table S6). Interestingly, this analysis highlighted motif associated with response to salicylic acid stimulus (GO:0,009,751). Moreover, some motifs were involved in circadian rhythm and response to auxin stimulus (Supplementary Table S6).

**Table 1:** The conserved motifs found in promoter regions of differentially expressed genes (DEGs) using the MEME analysis tool.

	Motif logo	E-value	Width	Best match in CIS-BP	Top significant BP terms identified by GOMO
Motif 1		7.10E−165	50	M06695	BP regulation of transcription
Motif 2		8.20E−143	29	M06719	BP regulation of transcription, DNA-dependent
Motif 3		1.20E−93	21	M06704, M06818, M06805, M06796	BP cytoskeleton organization BP regulation of transcription, DNA-dependent
Motif 4		1.00E−54	29	M01106, M06704, M01108, M06695	BP regulation of transcription
Motif 5		1.00E−38	37		
Motif 6		4.30E−31	29		
Motif 7		9.30E−36	29	M06704, M06805, M06695	BP regulation of transcription
Motif 8		2.90E−16	21	M06704, M06805, M06818	BP transmembrane receptor protein tyrosine kinase signaling pathway
Motif 9		1.30E−12	41		
Motif 10		1.60E−07	50		BP regulation of transcription
Motif 11		1.70E−06	15	M06719	BP leaf development BP regulation of transcription, DNA-dependent
Motif 12		5.00E−05	15	M06704	BP regulation of transcription
Motif 13		1.80E−04	20		BP regulation of transcription, DNA-dependent
Motif 14		2.10E−02	41		BP translation

### Identification of coexpression networks by WGCNA

**Figure 3:**
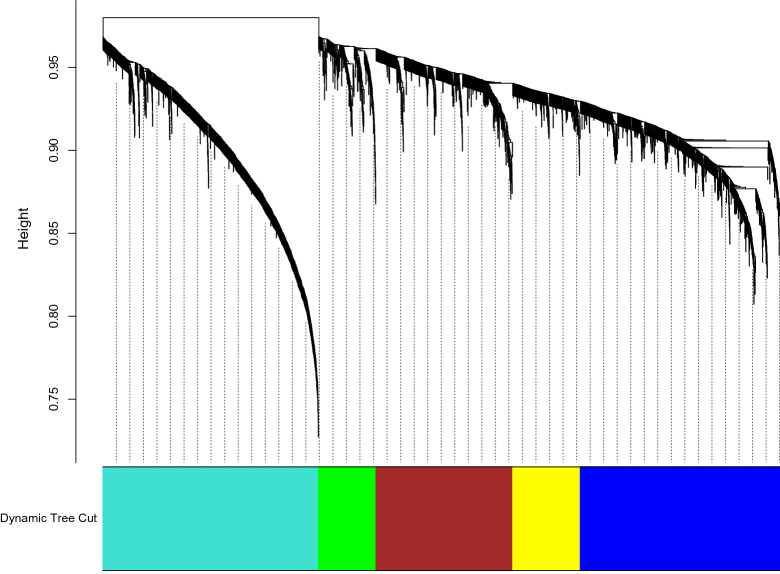
Weighted gene co-expression network analysis (WGCNA) of differentially expressed genes (DEGs). Cluster dendrogram showing co-expression modules identified by WGCNA. The modules are denoted in the colour bar.

We constructed gene coexpression networks using WGCNA based on the gene expression data of DEGs derived from the meta-analysis and feature selection. A total of 5 modules were detected that range from a maximum of 1145 genes of the turquoise module to the 305 genes of the green module (Fig. 3). The biological process gene ontology (GO) analysis revealed that the turquoise module was mainly related to small molecule metabolic process and microtubule cytoskeleton organization. Moreover, the 41 genes in the turquoise module were mainly related to protein transport. The genes in the blue module were mainly enriched in RNA biosynthetic process, whereas the genes in the brown module were mainly enriched in RNA splicing and response to reactive oxygen species. The yellow and green modules were also significantly enriched with the mRNA metabolic process and regulation of RNA metabolic process, respectively. The KEGG pathway analysis showed that phagosome and fatty acid degradation were significantly associated with turquoise and blue modules, respectively. Interestingly, genes in turquoise and green modules were significantly enriched in plant hormone signal transduction (Supplementary Table S7).

### Identification of hub genes

To discover the key genes associated with the modules, we identified genes with the most connections for each module that defended as hub genes (Supplementary Table S8, S9). The top 10 genes were screened from each module. For the turquoise module, the top hub was *CSLC5*-like (PtpAffx.214284.1.S1_at). The top hub genes in the blue module included amino acid transporter *AVT6A*-like (PtpAffx.7148.2.S1_a_at) and *MDAR6*-like (PtpAffx.27718.1.S1_s_at). In addition, *GIGANTEA*-like protein (PtpAffx.25624.1.A1_s_at), *E3 ligase Rma1H1*-like (PtpAffx.8582.3.S1_a_at) and *protein phosphatase 4* (PtpAffx.935.1.S1_at) were also hub genes in the brown, green and yellow modules, respectively (Fig. S1).

### Differential gene coexpression network analysis

**Figure 4:**
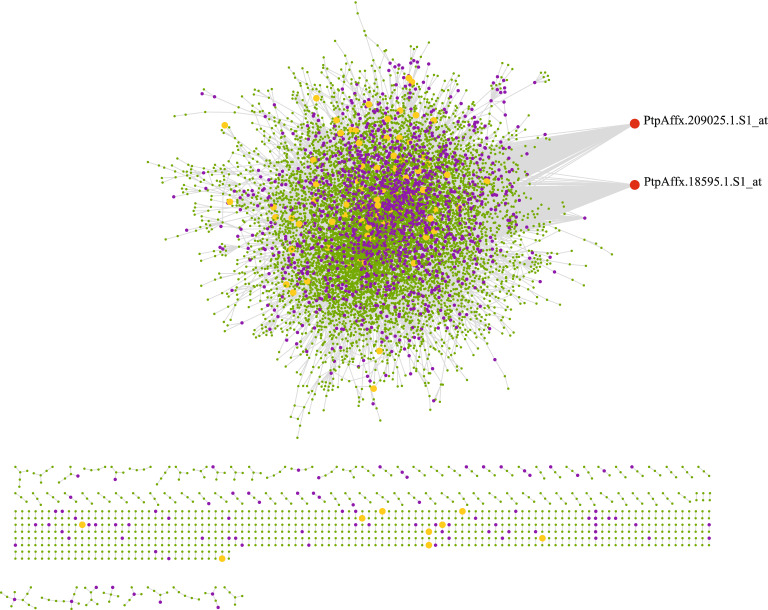
Differential co-expression network constructed using DiffCorr algorithm. Each node is a gene, violet nodes represent differentially expressed genes (DEGs) and red nodes represent genes with the most connectivity.

Generally, differential expression analysis investigates each gene separately and does not consider its relationship with other genes^28^. To detect changes in the patterns of expression of the genes between normal and stress conditions, we performed differential coexpression analysis and calculated Pearson's correlations of all gene pairs. We detected 28,863 gene pairs to have significant coexpression changes in the conditions at a cutoff of FDR < 0.01 (Supplementary Table S10). Finally, a differential correlation network with 11,344 nodes (genes) was constructed. In differential correlations between two networks, nodes with more connected edges represent genes that have the most distinct expression patterns between the two conditions. Acetyltransferase *NATA1*-like (PtpAffx.18595.1.S1_at) and *putative cytochrome P450* (PtpAffx.209025.1.S1_at) genes with 430 and 365 edges, respectively, were the top differentially connected genes (Fig. 4). Under normal conditions, 7705 of gene pairs, and under stress conditions, 8264 of gene pairs, respectively, showed a positive correlation that most of these genes related to response to abiotic stimulus and small molecule metabolic process. Additionally, 10,422 genes showed the opposite correlation direction between the two conditions. According to the GO analysis of the genes with different correlation directions, these genes are involved in response to stimulus and cellular catabolic process (Supplementary Table S11). The differential coexpression analysis revealed that *peptidylprolyl isomerase* (PtpAffx.25142.1.A1_at) and *thioredoxin-dependent peroxiredoxin* (PtpAffx.3462.1.A1_s_at) had the strongest change in correlation where correlation strength decreased from 0.67 to − 0.33 after stress.

## Discussion

Drought stress induces a huge range of responses that enable plants to survive in restriction of water availability. *Populus* is a woody plant model with sufficient genomic information, and due to the importance of this genus for biology studies, we collected comprehensive expression data for drought stress. We designed a pipeline with a systematic view for investigating transcriptional changes during drought stress to identify the genetic architecture in *Populus*. With meta-analysis and feature selection methods for expression data, we determined 3,594 genes that are differentially regulated by drought stress. Of these DEGs, 232 genes were identified by both methods and confirmed that many of the genes selected by feature selection methods were included among those selected by meta-analysis. The GO enrichment analysis suggests that DEGs are involved in a large range of biological functions. The majority of these GO terms were associations with metabolic process, response to stress, catabolic process. Both up-and down-regulated DEGs were enriched for the secondary metabolic process (Supplementary Table S3). However, more genes that belonged to this class were primarily up-regulated. The accumulation of secondary metabolites is an important part of a plant’s protection strategy against environmental stress^29^. The accumulation of phenolic and flavonoid compounds has been reported in *Populus* under drought stress^30^. A survey of KEGG pathways detected 17 pathways (Supplementary Table S3). The results revealed that the plant hormone signal transduction pathway was enriched. In this pathway, 17 DEGs such as *STK* (PTPAFFX.163630.1.S1_AT) and *ABF1* (PTPAFFX.215822.1.S1_S_AT) were upregulated. Plant hormones act as central integrators of signaling cascades in adaptation to stress^31^. A previous study demonstrated that ABA, ethylene, brassinosteroid and jasmonate signal pathways may be involved in *Populus tomentosa* cold response processes^32^. The *STK* and *ABF1* are associated with responses to drought stress in the plant^33^. The pathway analysis also demonstrated that drought stress had a significant effect on the carbon metabolism (Supplementary Table S3), indicating that *Populus* may has a defensive response through conserving energy in stress conditions. We also found that the expression changes of six genes related to the MAPK signaling pathway occurred during drought stress, which suggested that the MAPKs have key roles in the drought response in *Populus*. Among DEGs, transcription factors such as bZIPs, NACs and ERFs, which are known to be involved in stresses were also detected. The bZIP-type TFs play major roles in the regulation of development and salt stress responses of *Populus*^34^. Additionally, 24 members of the MYB family had a difference in expression between conditions (Fig. 2B). Previous studies have demonstrated that MYBs participate in various processes and have important functions in plant stress tolerance^35,36^. In *Tamarix hispida*, the overexpression of *ThMYB13* enhances salt stress tolerance^37^. These results imply a regulatory role of MYBs in *Populus* for drought response.

According to most feature selection models, two genes *ARF2* -like (PtpAffx.211941.1.S1_at) and *PYL4*-like (PtpAffx.31936.1.A1_at) defined as the important features (Supplementary Table S4) which are involved in several biological processes such as cellular metabolism and development regulation. ARF plays an important role in hormone regulation and plant stress response^38^. Recently, an *ARF* was discovered that is crucial for early developing xylem in *Populus*^39^. It suggests that the expression change of *ARF2* may be a reaction mechanism against water stress by impressing xylem function. In addition, The *PYL* genes are abscisic acid receptors that have a role in regulating drought tolerance^40^. A previous study showed that overexpression of *PYL4* in Arabidopsis resulted in drought resistance^41^.

To obtain further insights into the mechanism of gene regulation during drought stress in *Populus*, motifs and transcription factor binding sites were discovered in promoter regions of DEGs. We found 14 distinct conserved motifs comprised of ARID, C2H2, AHL, AP2/B3-like, BBR/BPC, MYB, and Sox (Table 1, Supplementary Table S5). The signal transduction, regulation of transcription and response to salicylic acid were important functional categories for these motifs (Supplementary Table S6). Previous studies showed the role of MYB in various biological processes, especially responses to environmental stresses^42^. The MYB binding site-*cis*-element is necessary for the gene expression of drought-inducible genes^43^. In *Populus*, the expression changes of MYB genes are observed under different stresses. These genes may also participate in the regulation of circadian rhythms, senescence-related ABA signaling cascade and cell-fate determination^44^. Yang et al.^45^ in their study detected *cis*-acting elements involved in abscisic acid and stress responsiveness in upstream regions of *PtrMYB053* and *PtrMYB081*. The AHL is a group of transcriptional regulators with a highly conserved structure in the plant kingdom and extensive information demonstrates function in plant growth and development as well as stress responses^46^. A previous study identified 37 *AHL* genes in *Populus trichocarpa* that expression levels of these genes were induced by drought stress^47^.

To assess the interactions among DEGs, a WGCNA was performed that can help to better understand the mechanisms involved in drought stress. The DEGs were grouped into 5 modules (Fig. 3). Functional analysis results showed that the genes in the modules were mostly associated with molecule metabolic process, RNA biosynthetic process, RNA splicing, and stress response (Supplementary Table S7). Some DEGs included in the turquoise module belonged to a generation of precursor metabolites and energy, and transport that reflecting their importance in response to stress. The saving of energy and maintenance of homeostasis under stress is considered an adaptive feature. During stress, the plant through adjustments of metabolism and gene expression shunt energy sources from processes of growth to adaptation^48^. The role of transport as a key regulatory molecule in sugar metabolism, abscisic acid signaling, stress responses and enhancing photosynthetic activity has been established^49,50^. The turquoise module included an *ABC*-2 *type transporter* (PtpAffx.85773.1.S1_at) and *TRH1* (PTP.121.1.S1_S_AT). ABC transporters are necessary for the internal detoxification of ions and act as a regulator for maintaining plant homeostasis under environmental stresses^51^. Previous studies have proposed that *TRH1* is required for root hair elongation and participates in auxin translocation and accumulation^52,53^.

Genes in the brown module were involved in RNA splicing and response to reactive oxygen species. Environmental stresses damage DNA and affect its stability. Studies demonstrate the importance of alternative splicing in DNA repair for plants^54^. Among genes in response to reactive oxygen species, we identified two *HSP15*- like (PTPAFFX.53720.1.S1_AT) and *APX3*- like (PTP.4040.1.S1_S_AT). Heat shock proteins are ubiquitous that accumulate in response to abiotic stress^55,56^. In *Populus euphratica*, HSPs had high transcript levels after stress^57^. In our results, *HSP15*-like (PTPAFFX.53720.1.S1_AT) significantly induced under drought stress. The yellow module genes enriched for vacuole organization. Vacuoles play a major function in plant trafficking pathways and response to environmental signaling and participate in oxidative-stress resistance^58^.

We also identified the gene with the highest connectivity (hubs) within each module (Supplementary Table S9) which are the key components of the networks. We found that the hub genes were enriched in the response to misfolded protein. The highly connected gene in the turquoise module, including *CSLC5*-like (PtpAffx.214284.1.S1_at) has been reported to be related to developing cell walls^59^. It has been shown that activation of *CSLC5*-like is essential for protection from adverse environmental conditions. The hub gene of the blue module (*protein phosphatase 4*) was involved in a variety of cellular functions^60^, suggesting a change in phosphorylation status is one of the most important regulatory ways to stress responses in all *Populus*. Interestingly, amino acid transporter *AVT6A*-like (PtpAffx.7148.2.S1_a_at) and *PLATZ1* (PtpAffx.27718.1.S1_s_at) were the most interconnected genes in the blue module. *PLATZ* encodes a class of plant-specific zinc-finger transcription factor that functions in plant growth, development and abiotic stresses through ABA^61^. Among the hub genes in the blue module, *uspA*-like (Ptp.4430.1.S1_a_at) is of interest. Universal stress proteins (USP) can arrest cell growth and constitute a biological defense mechanism in stress conditions^62^. In particular, *GIGANTEA*-like protein (PtpAffx.25624.1.A1_s_at) was identified as one of the hub genes in brown module. This gene functions in developmental stage transitions and stress responses. Recent findings exhibit that *GIGANTEA*-like protein mediates circadian rhythm and responses to stress in poplar^63^.

To identify significant changes in the coexpression structure and provide an overview of gene expression interactions between normal and stress conditions, we performed differential gene coexpression analysis. We identified over 28,000 gene pairs whose expression levels were significantly correlated. We obtained a network with 11,344 genes (Fig. 4, Supplementary Table S10) which among them, 1460 genes were DEGs. Genes *Acetyltransferase NATA1*-like (PtpAffx.18595.1.S1_at) and *putative cytochrome P450* (PtpAffx.209025.1.S1_at) had most connections in the network. *NATA1* has implicated the regulatory function of polyamine acetylation and plays a function in the complex cross-talk between salicylate and jasmonate signaling^64^. A previous study indicated that *NATA1* is also involved in ABA-mediated stomatal closure in *A. thaliana*^65^. Cytochrome P450s are also one of the largest enzyme families and play an essential role in stress response. These key enzymes have also been detected in *Populus trichocarpa* that participate in the volatile formation^66^.

The results also indicated that the coexpression between *peptidylprolyl isomerase* (PtpAffx.25142.1.A1_at) and *thioredoxin-dependent peroxiredoxin* (PtpAffx.3462.1.A1_s_at) genes was extensively changed after stress. The peptidylprolyl isomerase was found associated with the folding of newly synthesized proteins in the cellular processes^67^. Protein folding is a fundamental process for cell survival under adverse environmental conditions^68^. It was suggested that peptidylprolyl isomerases have a role in abiotic stress response in plants^69^. In addition, thioredoxin-dependent peroxiredoxin as part of antioxidant defenses and redox signaling is essential for cellular response against oxidative stress^70^. Previous observation implies a possible link between mitochondrial thioredoxin system and peptidyl prolyl *cis*–trans isomerase activity^71^.

Collectively, in this study, we implemented a strategy for the detection of genes and underlying mechanisms involved in response to drought stress in *Populus*. Based on the meta-analysis, feature selection algorithms and coexpression analysis, we highlighted genes and pathways that play key roles during drought stress. The findings revealed that transcriptional changes under drought stress can be extremely varied in *Populus*. These results showed that drought stress induces a complex of hormone signaling pathways. In addition, TFs such as MYBs and bZIPs are key for *Populus* response. In particular, our analysis suggests that *ARF2*-like (PtpAffx.211941.1.S1_at) and *PYL4*-like (PtpAffx.31936.1.A1_at) genes can be potential candidates for screening and breeding purposes in *Populus*. The results obtained can pave the way for understanding the molecular basis of drought response and for further investigations in *Populus*.

## Methods

### Data collection and preprocessing

The array expression datasets were retrieved from the Gene Expression Omnibus (GEO) (https://www.ncbi.nlm.nih.gov/gds) and the ArrayExpress (https://www.ebi.ac.uk/arrayexpress) (Supplementary Table S1). The data consisted of 13 studies that generated from the Affymetrix GeneChip Poplar Genome Array. The chip description file (CDF) and annotation file were downloaded from the Affymetrix site. The robust multichip average (RMA) algorithm was used to background corrected and normalized gene expression data^72^ from the Affy R package. Moreover, genes that had low mean and variation in expression values were filtered out. Finally, an empirical Bayes method was performed to correct non-biological differences and remove batch effects from gene expression datasets using ComBat function in the SVA R package^73^.

### Differential gene expression analysis

To identify upregulated and downregulated differentially expressed genes (DEGs) among normal and drought samples, meta-analysis was performed using the effect size combination method in the metaMA R package^74^. Genes with an FDR ≤ 0.05 were defined as DEGs.

Feature selection algorithms were employed to reduce the dimensionality of the expression dataset and identify the gene expression features between normal and stress conditions. We implemented various attribute weighting algorithms, including Support Vector Machine (SVM), Chi Squared, Information Gain, Information Gain Ratio, Deviation, Gini Index, Uncertainty, Relief, and PCA on corrected data to identify the most important genes^75^. The feature selection was performed using RapidMiner Studio software (version 7.0.001).

### Functional analysis

To investigate the functions related to the DEGs, we conducted the Gene Ontology (GO) using the AgriGO (http://systemsbiology.cau.edu.cn/agriGOv2/). The web server REVIGO was used to remove redundant enriched GO terms. The Kyoto Encyclopedia of Genes and Genomes (KEGG) pathways enrichment analysis was performed by gProfiler tool (https://biit.cs.ut.ee/gprofiler/gost). GO terms and KEGG pathways with an adjusted *P*-value < 0.05 were defined as significant. In addition, transcription factors (TFs) families were obtained by BLASTX search against *Populus trichocarpa* transcription factors (http://planttfdb.cbi.pku.edu) with a cut-off of E ≤ 10^−6^.

### *Cis*-elements analysis

To discover conserved motifs and investigate *cis*-elements in DEGs, the 1kbp upstream sequences of genes were extracted from Ensembl Plants (http://plants.ensembl.org) and then were submitted to a search by the MEME program (http://meme-suite.org/). The Tomtom v 5.4.1 tool (http://meme-suite.org/tools/tomtom)^76^ was employed for the identification of known TF binding sites based on the CIS-BP database. The GoMo tool (http://meme-suite.org/tools/gomo) was also used for the biological function of motifs.

### Coexpression network analysis and identification of potential hub genes

The weighted gene correlation network analysis (WGCNA)^77^ was used to construct the co-expression network and identify gene modules. First, the gene expression similarity matrix was built based on normalized gene expression data of the DEGs by determining Pearson’s correlation coefficient (PCC) between gene pairs. Subsequently, the gene expression similarity matrix was transformed into an adjacency matrix using an appropriate soft threshold value (β) of 10. Then, the adjacency matrix was converted into a topological matrix by the topological overlap matrix (TOM) by the TOM similarity algorithm. Finally, the dynamic tree-cut algorithm method was used to identify the modules of co-expressed DEGs with the maxBlockSize of 6,000, minModuleSize of 30 and mergeCutHeight of 0.2. In addition, according to gene intramodular connectivity, genes with a high kME were considered hub genes in each module. The gProfiler tool was used to perform GO and KEGG functional enrichment analysis of the significant modules. We used the cystoscope to visualize the networks.

### Differential coexpression analysis

**Figure 5:**
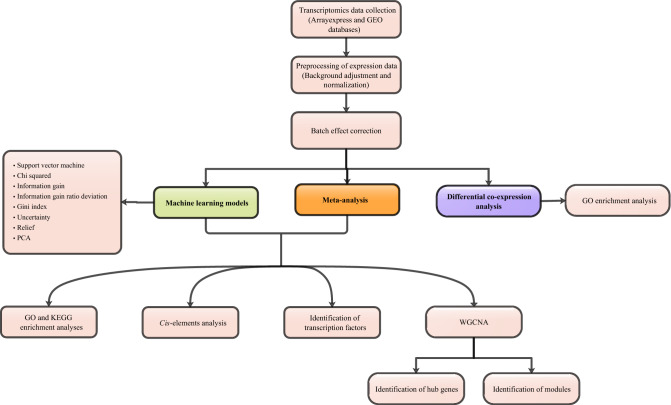
A schematic overview of the multistep strategy for understanding aspects of the response of *Populus* to drought stress.

To identify significant differential correlations between normal and stress conditions, a differential coexpression gene network was constructed using the DiffCorr R package^78^. First, the expression data were divided into normal and stress groups, and then genes with zero expression or low variation across samples were removed that resulting in 30,865 genes selected for further analysis. Pearson correlations were calculated between each pair of genes for each group and each correlation value was transformed to *Z* score using Fisher’s *Z* transformation. Next, the difference in correlation of a gene pair between normal and stress conditions was calculated by the equation: $\Delta \mathrm{Z}=\frac{{Z}_{S}-{Z}_{N}}{\sqrt{\frac{1}{{n}_{\mathrm{S}}-3} + \frac{1}{{\mathrm{n}}_{\mathrm{N}}-3}}}$ where *n*_*N*_ and *n*_*S*_ are respectively the numbers of samples under normal and stress conditions. Finally, Fisher’s *z*-test was applied to detect significant differential correlations. Differential correlations with a FDR < 0.01 were considered significant^79^. The differential coexpression network was visualized by Cytoscape. A schematic workflow summarizing the major steps of this study is shown in Fig. 5.

## Supplementary Information

Supplementary Information 1.

Supplementary Information 2.

## Data Availability

All data generated or analysed during this study are available from the corresponding authors on reasonable request.
