# Publisher Correction: Integration of meta‑analysis, machine learning and systems biology approach for investigating the transcriptomic response to drought stress in *Populus* species

**DOI:** 10.1038/s41598-023-30033-z

**Published:** 2023-02-22

**Authors:** Ahmad Tahmasebi, Ali Niazi, Sahar Akrami

**Affiliations:** Institute of Biotechnology, Shiraz University, Shiraz, 7144165186 Iran

Correction to: *Scientific Reports* 10.1038/s41598-023-27746-6, published online 16 January 2023

The original version of this Article contained an error in the order of the Figures. Figures 1, 2, 3, 4 and 5 were published as Figures 5, 2, 3, 4 and 1. The Figures appear in the incorrect order below.

**Figure 1:**
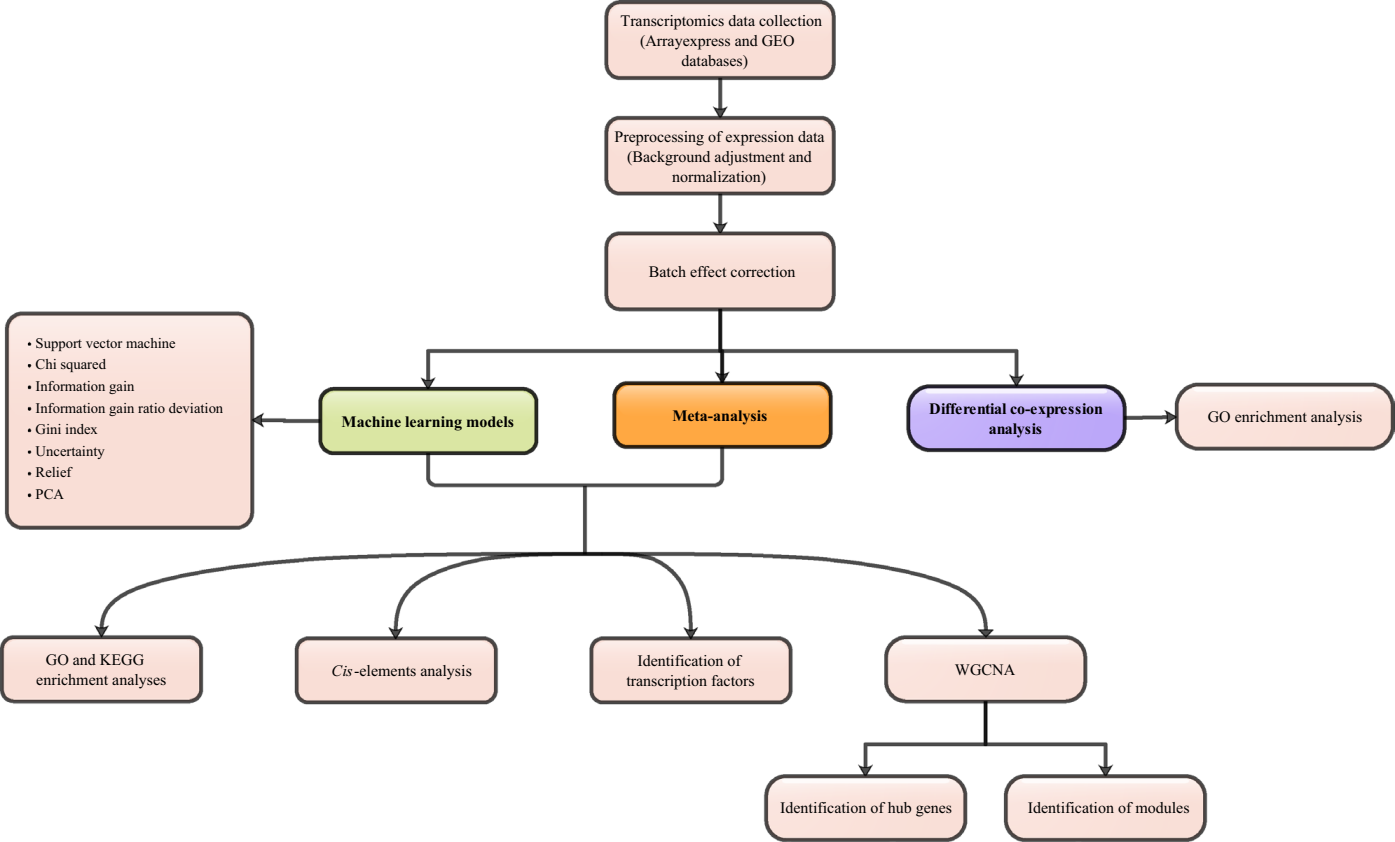
A schematic overview of the multistep strategy for understanding aspects of the response of Populus to drought stress.

**Figure 2:**
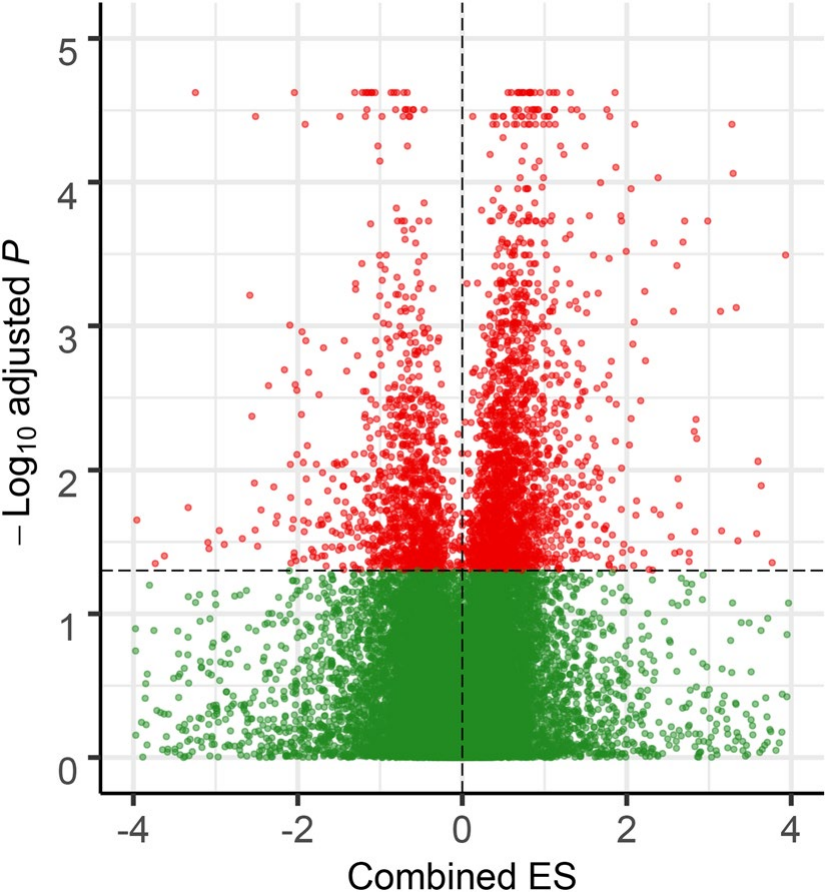
Gene expression comparison between normal and stress conditions. A volcano plot showing combined effect size (x-axis) and significance level (− log10-adjusted p-value; y-axis) for genes differentially expressed between normal and stress samples. The significant up and down-regulated genes are plotted as red dots.

**Figure 3:**
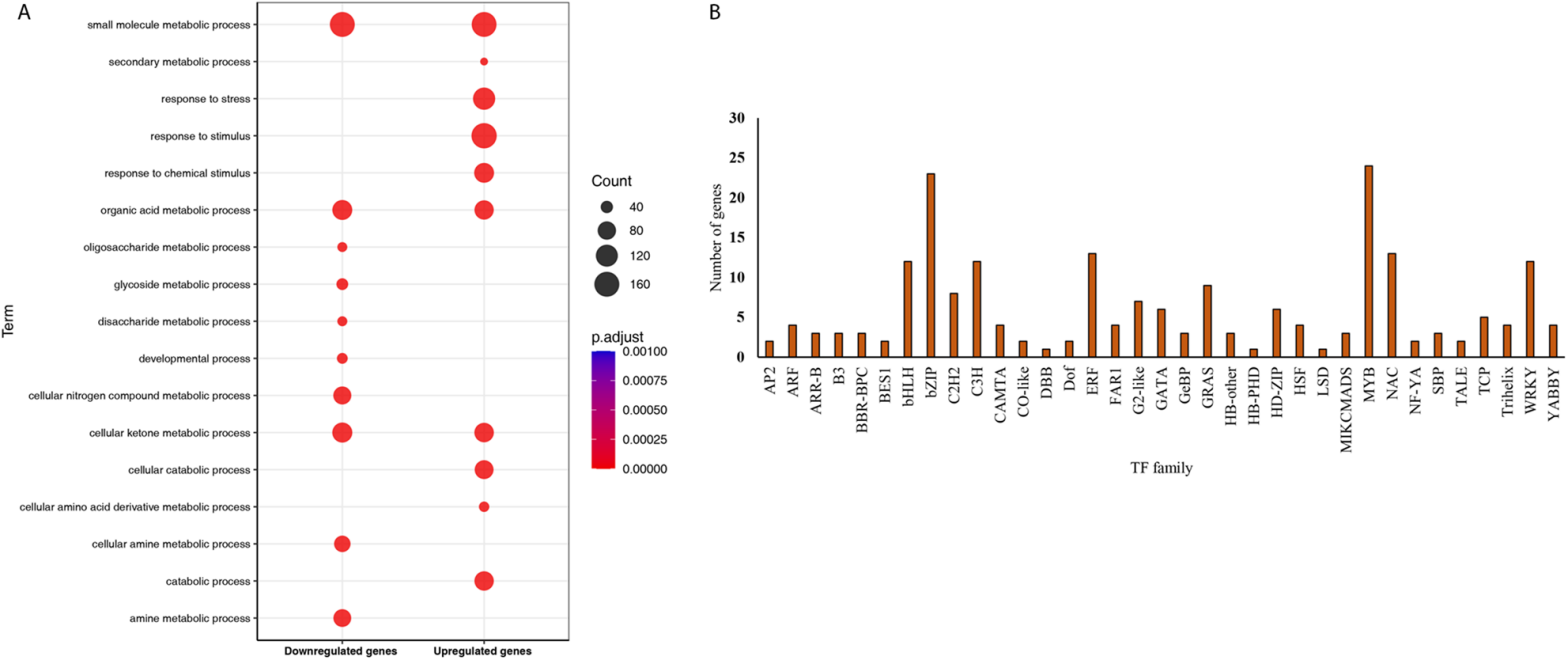
(**A**) Top 10 gene ontology biological processes (GO-BP) terms of the up-regulated and the down regulated differentially expressed genes (DEGs). (**B**) Distribution of transcription factor (TF) families in the differentially expressed genes (DEGs). The number of genes is shown for each transcription factor family on the y-axis.

**Figure 4:**
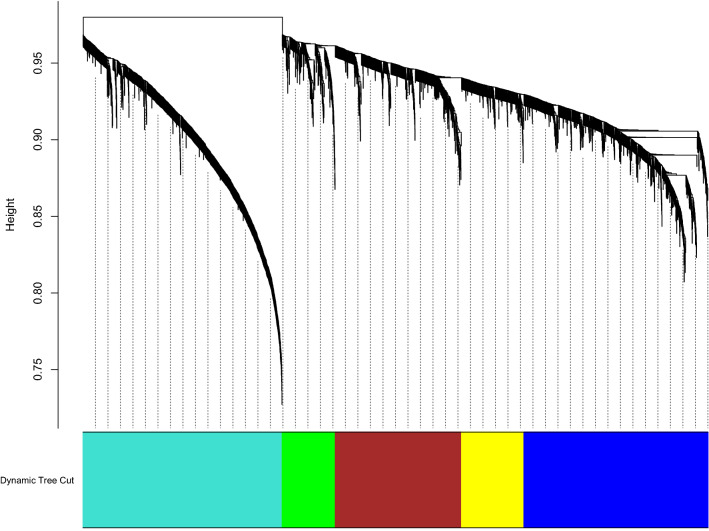
Weighted gene co-expression network analysis (WGCNA) of differentially expressed genes (DEGs). Cluster dendrogram showing co-expression modules identified by WGCNA. The modules are denoted in the colour bar.

**Figure 5:**
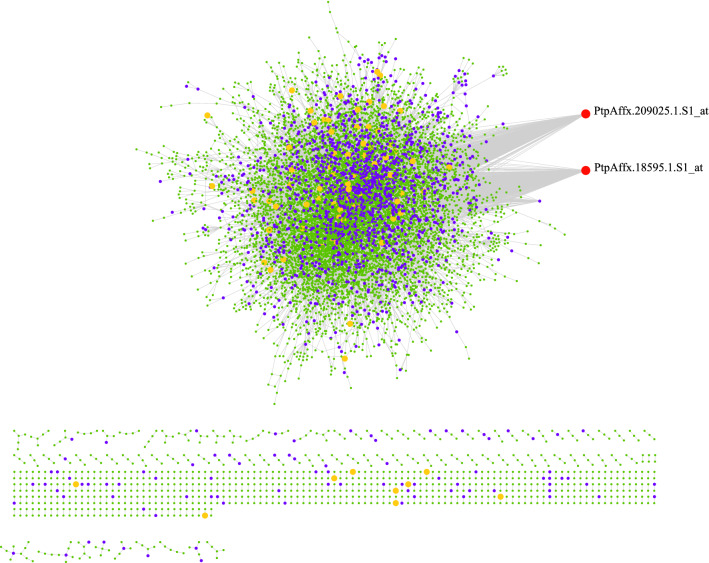
Differential co-expression network constructed using DiffCorr algorithm. Each node is a gene, violet nodes represent differentially expressed genes (DEGs) and red nodes represent genes with the most connectivity.

The original Article has been corrected.

