# Supplementary figures and images for: Integration of meta-analysis, machine learning and systems biology approach for investigating the transcriptomic response to drought stress in Populus species
Source: Sci Rep. 2023 Jan 16;13:847. doi: 10.1038/s41598-023-27746-6 (PMC9842770; doi:10.1038/s41598-023-27746-6)

**A**

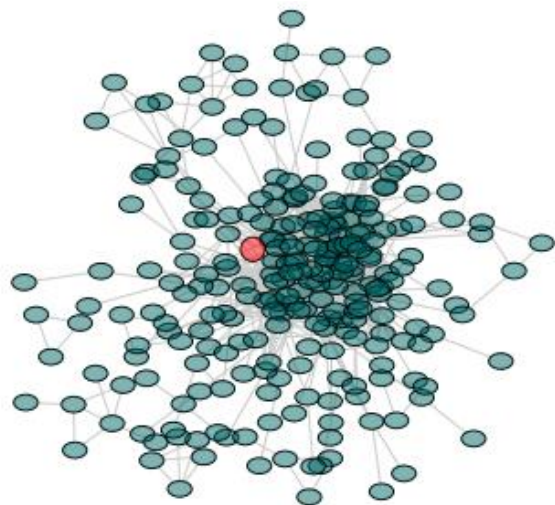

**B**

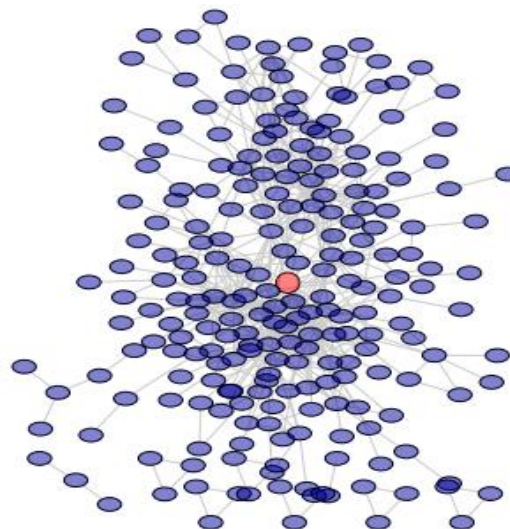

**C**

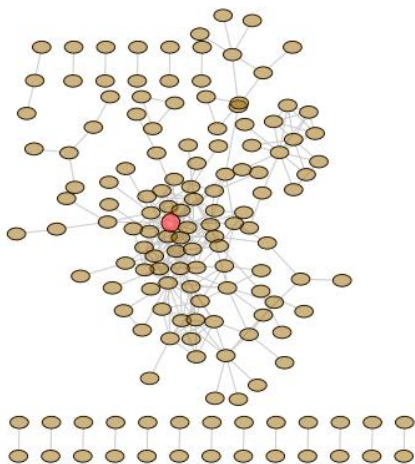

**D**

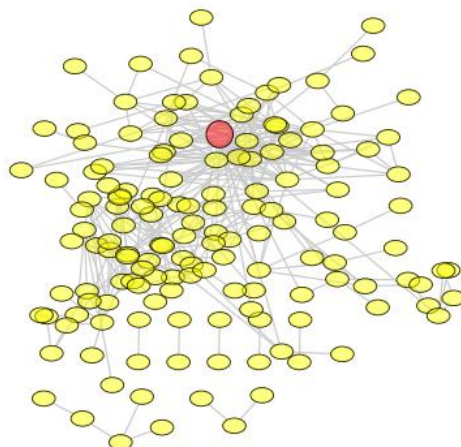

**E**

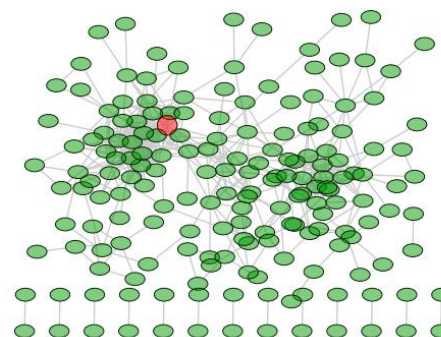

Supplement: Supplementary file 1 — Supplementary Information 1. [file 41598_2023_27746_MOESM1_ESM.pdf]
